# Integration of transcriptomic, proteomic, and metabolomic data to identify lncRNA rPvt1 associations in lipopolysaccharide-treated H9C2 cardiomyocytes

**DOI:** 10.3389/fgene.2023.1278830

**Published:** 2023-11-29

**Authors:** Tie-Ning Zhang, Ri Wen, Yu-Hang Yang, Ni Yang, Chun-Feng Liu

**Affiliations:** Department of Pediatrics, PICU, Shengjing Hospital of China Medical University, Shenyang, China

**Keywords:** lncRNA, metabolomics, proteomics, rPvt1, sepsis, transcriptomics

## Abstract

**Background:** Recent evidence has shown that the long non-coding RNA (lncRNA) rPvt1 is elevated in septic myocardial tissues and that its knockdown attenuates sepsis-induced myocardial injury. However, the mechanism underlying the role of rPvt1 in septic myocardial dysfunction has not been elucidated.

**Methods:** In this study, we performed transcriptomic, proteomic, and metabolomic assays and conducted an integrated multi-omics analysis to explore the association between rPvt1 and lipopolysaccharide (Lipopolysaccharide)-induced H9C2 cardiomyocyte injury. LncRNA rPvt1 silencing was achieved using a lentiviral transduction system.

**Results:** Compared to those with the negative control, rPvt1 knockdown led to large changes in the transcriptome, proteome, and metabolome. Specifically, 2,385 differentially expressed genes (DEGs), 272 differentially abundant proteins and 75 differentially expressed metabolites (DEMs) were identified through each omics analysis, respectively. Gene Ontology functional annotation, Kyoto Encyclopedia of Genes and Genomes, Nr, eukaryotic orthologous groups, and Clusters of Orthologous Groups of Proteins pathway analyses were performed on these differentially expressed/abundant factors. The results suggested that mitochondrial energy metabolism might be closely related to the mechanism through which Pvt1 functions.

**Conclusion:** These genes, proteins, metabolites, and their related dysregulated pathways could thus be promising targets for studies investigating the rPvt1-regluatory mechanisms involved in septic myocardial dysfunction, which is important for formulating novel strategies for the prevention, diagnosis and treatment of septic myocardial injury.

## Introduction

Sepsis is a systemic inflammatory syndrome initiated by a bacterial infection, leading to multiple organ dysfunction, especially in the cardiovascular system (L'Heureux et al., 2020; Joffre et al., 2020). The clinical risk of cardiovascular events in survivors of severe sepsis is relatively high (Yende et al., 2014). Myocardial dysfunction caused by sepsis is characterized by decreased systolic contractility and impaired diastolic function and is an important contributor to septic shock (Walley, 2018). Although numerous studies have investigated septic myocardial dysfunction, its underlying mechanisms remain largely unknown.

Lipopolysaccharide (LPS) is a major component of the outer membrane of most Gram-negative bacteria (Giordano et al., 2020). Further, it is a common endotoxin that activates epithelial, endothelial, and mononuclear macrophages *via* signal transduction systems (White and Demchenko, 2014). Studies have shown that LPS causes septic myocardial injury by promoting myocardial cell inflammation, injury, and apoptosis (Li et al., 2019; Li et al., 2021). Accordingly, LPS-induced myocardial cell injury comprises a common cellular model for simulating septic cardiac dysfunction *in vitro* (Haileselassie et al., 2019; Li et al., 2022).

Recently, long noncoding RNAs (lncRNAs) have been investigated in studies of septic myocardial dysfunction because of their epigenetic regulatory functions (Piccoli et al., 2015; Liu and Chong, 2021). LncRNAs comprise a group of non-protein-coding transcripts containing more than 200 nucleotides, and they account for 80%–90% of the human genome and play pivotal roles in epigenetic regulation (Hombach and Kretz, 2016; García-Padilla et al., 2022). The functions of several lncRNAs (Sun et al., 2020; Xing et al., 2020; Ni et al., 2021) in septic cardiomyopathy have been previously discussed. Among them, the lncRNA Pvt1 has attracted our attention, as it is a novel oncogene in multiple cancers (Chen et al., 2019; Zhou et al., 2020). In several injurious diseases, lncRNA Pvt1 can promote oxidative stress, inflammation, and cell damage (Liu et al., 2021; Guo et al., 2021). Guo et al. demonstrated that lncRNA Pvt1 was elevated in the heart tissue of LPS induced sepsis mice, and Pvt1 aggravates LPS-induced myocardial injury by promoting M1 polarization (Luo et al., 2021). Also, lncRNA Pvt1 was markedly upregulated in myocardial ischemia-reperfusion tissues as well as hypoxia/reoxygenation-induced H9C2 cells, and its knockdown ameliorates the myocardial damage (Li et al., 2021). Intriguingly, in our previous work, we also identified that the *Rattus norvegicus* lncRNA Pvt1 (named as lncRNA rPvt1, gene ID: ENSRNOG00000062170; transcript ID: ENSRNOT00000092896) was among the significantly upregulated lncRNAs in the left ventricular tissues of rats suffered septic shock, and knockdown of rPvt1 influenced LPS-induced cardiomyocyte apoptosis (Zhang et al., 2019). These findings imply that Pvt1 may be a potential target for septic cardiac dysfunction. However, the mechanism underlying the function of rPvt1 has not been clarified.

With the development and application of high-throughput technology, increasing biomedical studies have adopted integrated multi-omics results to investigate disease pathogenesis and therapeutic mechanisms. Owing to this, researchers can obtain information from omics data at different molecular levels, such as the genome, transcriptome, proteome, epigenome, metabolome, liposome, and microbiome (Sun and Hu, 2016; Kang et al., 2022). The integration of multi-omics analysis has thus revolutionized biology, providing a deep and advanced understanding of biological processes and molecular mechanisms. In this study, we established an LPS-induced cardiomyocyte injury model and treated the cells with a lentiviral vector system to knock down lncRNA rPvt1 expression. Subsequently, we conducted transcriptomic, proteomic, and metabolomic analyses to investigate potential lncRNA rPvt1 associations during this process.

## Materials and methods

### Construction of lentiviral particles

Lentivirus-producing cassettes were purchased from Beijing Syngentech Co., Ltd. (Beijing, China), and lentivirus preparation was also performed by this company. Briefly, a short hairpin RNA (shRNA) targeting the CCT​ATG​AGG​TGA​TGA​TAA​A sequence of Pvt1 was constructed and inserted into the lentiviral expression vector pLV-hU6-NC shRNA01-hef1a-mNeongreen-P2A-Puro. The sequence of the negative control shRNA was AAA​CGT​GAC​ACG​TTC​GGA​GAA​CGA​ATT​CTC​CGA​ACG​TGT​CAC​GTT​T. For the production of lentiviral particles, HEK293FT cells were pre-seeded and co-transfected with a lentivirus packaging vector, transfer plasmid, and the constructed lentiviral expression vector. Transfected cells were cultured for 48 h to produce lentiviral particles.

### Cell culture, lentivirus infection, and cell treatment

Rat H9C2 cardiomyocytes were obtained from the Shanghai Institute of Biochemistry and Cell Biology (Shanghai, China). Cells were cultured in Dulbecco’s modified Eagle’s medium (Gibco, Grand Island, NY, United States) containing 10% fetal bovine serum (Hyclone, Logan, UT, United States) at 37°C in a humidified incubator with 95% air and 5% CO_2_. After reaching 80% confluence, the cells were infected with lentiviral particles at an MOI of 30 and cultured for 96 h. The cells were then treated with 10 μg/mL of LPS that derived from *Escherichia coli* O55:B5 (LPS 055: B5, L2880, Sigma) for 24 h (Lei et al., 2018; Li et al., 2019).

### Reference-based transcriptomic analysis

Total RNA was quantified using the Qubit™ RNA detection kit (Thermo Fisher Scientific, Waltham, MA, United States). Sequencing libraries were constructed according to the protocols of the Hieff NGS™ MaxUp Dual-mode mRNA Library Prep Kit for Illumina^®^ (Yeasen Biotechnology Co., Ltd. Shanghai, China). Briefly, RNA was enriched with oligo (dT) magnetic beads and fragmented using the Frag/Prime buffer. The fragmented RNA was transcribed into cDNA, then the cDNA was then purified using Hieff NGS DNA Selection Beads (Yeasen). Purified cDNA was end-repaired, followed by the addition of a poly(A) sequence and ligation into the adapters. Fragments of the desired size were selected through agarose gel electrophoresis. The constructed libraries were sequenced on an Illumina HiSeq™ platform (Illumina, San Diego, CA, United States).

Raw image files generated using the Illumina HiSeq™ platform were converted into raw reads using CASAVA base calling. Raw reads were cleaned using the Trimmomatic software. Adapters and reads with low quality (>5% uncertain bases) were filtered out, and clean reads were obtained. HISAT2 software was used to map clean reads to the reference genome. To quantify the gene expression levels, transcripts per million (TPM) values were calculated using StringTie. The correlation between the samples was determined based on Pearson’s correlation analysis. Differentially expressed genes (DEGs) were identified using the DESeq R package. The threshold for significant differential expression was a q < 0.05 and log_2_|fold-change|>1. DEGs were functionally annotated based on a comparison with entries in the Gene Ontology (GO), Kyoto Encyclopedia of Genes and Genomics (KEGG), and Nr eukaryotic orthologous groups (KOG) databases.

All images were obtained using R language. The TPM density curve, principal component analysis (PCA) diagram, and histograms of GO, KEGG, and KOG enrichment results were plotted using ggplot2. The Venn diagram was plotted using plotrix. Heatmaps of Pearson’s correlation and differential expression were plotted using pheatmap. GO and KEGG networks were plotted using Cytoscape.

### Four-dimensional label-free quantitative proteomic analysis

Total protein in the cells was quantified using a BCA kit (Beyotime Institute of Biotechnology, Shanghai, China) according to the manufacturer’s instructions. The protein was digested with trypsin into peptides, which were dissolved in solvent A (0.1% formic acid, 2% acetonitrile in water) and loaded onto a homemade reversed-phase analytical column. Peptides were separated using a gradient of solvent B (0.1% formic acid in 90% acetonitrile). The separated peptides were subjected to a capillary source followed by timsTOF Pro (Bruker Daltonics) mass spectrometry in parallel accumulation serial fragmentation mode. The applied electrospray voltage was 1.60 kV, the m/z scan range was 100–1,700 m/z, and the dynamic exclusion was set to 30 s.

Original MS/MS data were processed using the MaxQuant search engine. Tandem mass spectra were searched against the Rattusnorvegicus_10116_PR_20210721 database concatenated with the reverse decoy and contaminant databases. The enzyme digestion mode was set to trypsin/P, the number of missing cuts was set to two, and the minimum length of the peptide was set to seven amino acid residues. The maximum number of peptide modifications was set to five. The mass error tolerance for primary parent ions was set to 20 ppm for all of the first and main searches and the secondary fragment ions. The FDR was adjusted to <1%. The threshold for significant differential abundance was a *p* < 0.05 and fold-change>1.5 or <1/1.5. The differentially abundant proteins (DAPs) were functionally annotated based on a comparison with entries in the GO, KEGG, and clusters of orthologous groups of protein (COG) databases. In addition, a protein–protein interaction (PPI) network was constructed using the Search Tool for the Retrieval of Interacting Genes database.

All images were obtained using the R language. The protein distribution chart, PCA diagram, and histograms of GO, KEGG, and COG enrichment results were plotted using ggplot2. The heatmap for differential abundance was plotted using pheatmap. A pie chart of the subcellular localization was plotted using the R function Pie. The PPI network was plotted using Cytoscape software.

### Widely targeted metabolomic analysis

Cell samples were mixed with a methanol–water (4:1, v/v) solution containing an internal standard and vortexed. The samples were subjected to three freeze–thaw cycles and centrifuged to collect the supernatant. Cellular metabolites were analyzed using an ultra-performance liquid chromatography (UPLC)-electrospray ionization (ESI)-tandem mass spectrometry (MS/MS) system.

Based on a self-generated database (Metware Database), metabolite information of the samples, including the Q1 precise molecular mass, secondary fragmentation, retention time, and isotope peak, was matched to the database for qualitative analysis. Quantitative metabolite analyses were conducted using the multiple reaction monitoring mode with the triple quadrupole (QQQ) mass spectrometer.

Metabolite data were analyzed using Analyst 1.6.3 software. Based on the results of orthogonal partial least squares-discriminant analysis, variable importance in projection (VIP) was analyzed using multivariate analysis. The threshold for significant differentially expressed metabolites (DEMs) was VIP≥1 and log_2_|fold-change|>1. The correlation among the DEMs was calculated using Pearson’s analysis, and the enrichment of DEMs was annotated based on a comparison with entries in the KEGG database.

### Energy-targeted metabolomic analysis

Energy-targeted metabolomics involves 68 important metabolites, including amino acids, carbohydrates, coenzymes, vitamins, nucleotides, organic acids and their derivatives, phosphate sugars, and phosphoric acids. Standards for these metabolites were used for accurate quantification. Energy-targeted metabolomic analysis was conducted using a UPLC-ESI-MS/MS system. Metabolite data analysis was conducted using MultiQuant 3.0.3 software. The threshold for significant differential energy metabolites was VIP≥1 and a fold-change>1.5 or <1/1.5. The DEMs were functionally annotated based on a comparison with entries in the KEGG databases.

All images of the metabolomic study were acquired using R language. A Pearson’s correlation heatmap was plotted using corrplot. Z-score charts and KEGG enrichment histograms were plotted using ggplot2 software.

### Correlation analysis between transcriptomic and proteomic data

The RNA information obtained from the transcriptomic analysis and protein information obtained from the proteomic analysis were matched and integrated. The DEGs and DAPs were mapped based on standard NCBI Gene Symbols, and overlapping genes were identified using a Venn diagram. Transcriptomic and proteomic GO/KEGG association analyses were performed to compare the similarities of altered genes at both the RNA and protein levels. Histograms of the GO and KEGG enrichment results were plotted using the R package ggplot2.

### Correlation analysis between proteomic and metabolomic data

The protein information obtained from the proteomic analysis and metabolite information obtained from the metabolomic analysis were matched and integrated. The DAPs and DEMs in the comparison group were subjected to correlation analysis and mapped simultaneously to the KEGG database. All images were obtained using R language. A Pearson’s correlation heatmap was plotted using corrplot. A nine-quadrant diagram was plotted using dplyr. A loading plot diagram of DAPs and DEMs was constructed using OmicsPLS. The DAP–DEM network was plotted using Cytoscape software, and the KEGG histogram was plotted using ggplot2.

### Correlation analysis between transcriptomic and metabolomic data

The RNA information obtained from the transcriptomic analysis and metabolite information obtained from the metabolomic analysis were matched and integrated. The DEGs and DEMs in the comparison group were subjected to expression correlation analysis and mapped simultaneously to the KEGG database. All images were obtained using R language. A Pearson’s correlation heatmap was plotted using corrplot. A nine-quadrant diagram was plotted using dplyr. A loading plot diagram of DEGs and DEMs was constructed using OmicsPLS. The KEGG histogram was plotted using ggplot2 software, and canonical correlation analysis (CCA) diagrams were plotted using mixOmics.

## Results

### Transcriptomic sequencing analysis of lncRNA rPvt1 associations in LPS-treated H9C2 cardiomyocytes

**FIGURE 1:**
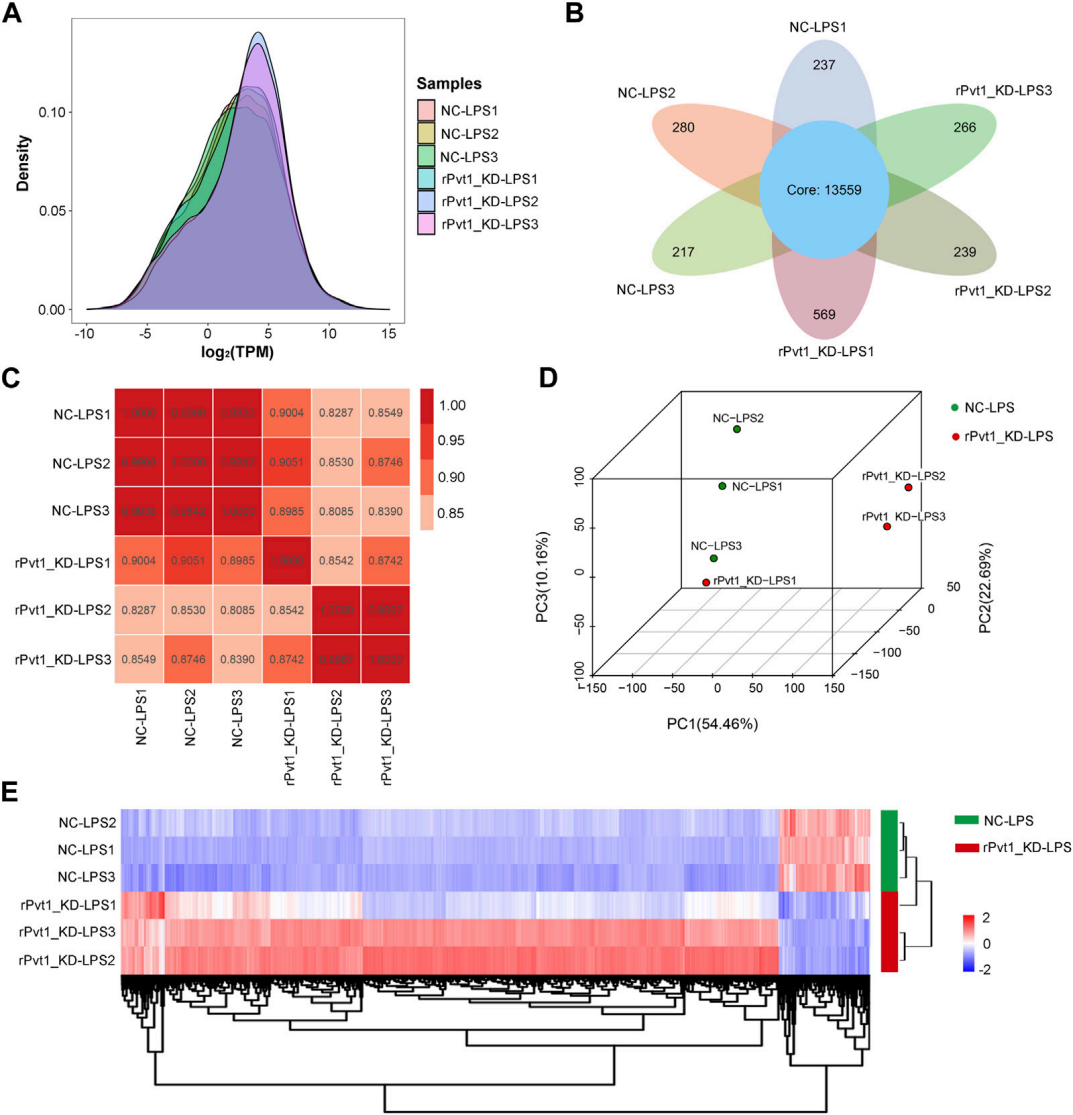
Transcriptomic sequencing analysis of lncRNA Pvt1 associations in LPS-treated H9C2 cardiomyocytes. Rat H9C2 cardiomyocytes were infected with NC-shRNA harboring or rPvt1-shRNA harboring lentiviral particles for 96 h then treated with 10 μg/mL of LPS for 24 h. Transcriptomic sequencing were established with three duplicated cell samples in each group. **(A)** TPM density distribution diagram of each sample. The *x*-axis represents the log_2_TPM of genes, and the *y*-axis represents the probability density. **(B)** Venn diagram of the identified genes in all sampless. **(C)** Heatmap of the Pearson’s correlations in terms of gene expression levels between samples. **(D)** PCA analysis based on the gene expression profile. **(E)** Heatmap of DEGs between NC-LPS group and rPVT1_KD-LPS group. Significantly upregulated and downregulated genes are colored in red and blue, respectively. Abbreviations: DEGs, differentially expressed genes; KD, knockdown; LPS, lipopolysaccharide; NC, negative control; PCA, principal component analysis; rPvt1, *Rattus norvegicus* lncRNA Pvt1; shRNA, short-harpin RNA; TPM, transcripts per million.

In the transcriptomic sequencing analysis, gene expression was calculated by counting the sequences (reads) in the genomic or gene exon regions. In this study, TPM values were used to calculate gene expression. By comparing the density distribution curves of each sample, we observed that the relative densities of most genes were consistent among the samples (Figure 1A). The Venn diagram showed that 13,559 genes were shared among all six samples (Figure 1B). In order to study the distribution and reproducibility of data between samples, correlation analysis and PCA analysis were carried out (Figures 1C, D). The results showed that the coefficients for the overall correlations between samples were all over 0.8, indicating a close gene expression pattern in both the groups and replicates. Accordingly, we identified 2,385 DEGs (2094 with upregulated and 291 with downregulated expression) based on significant changes in expression between the Lv-shNC-infected (NC-LPS) and Lv-shPvt1-infected (rPvt1_KD-LPS) groups (Figure 1E).

**FIGURE 2:**
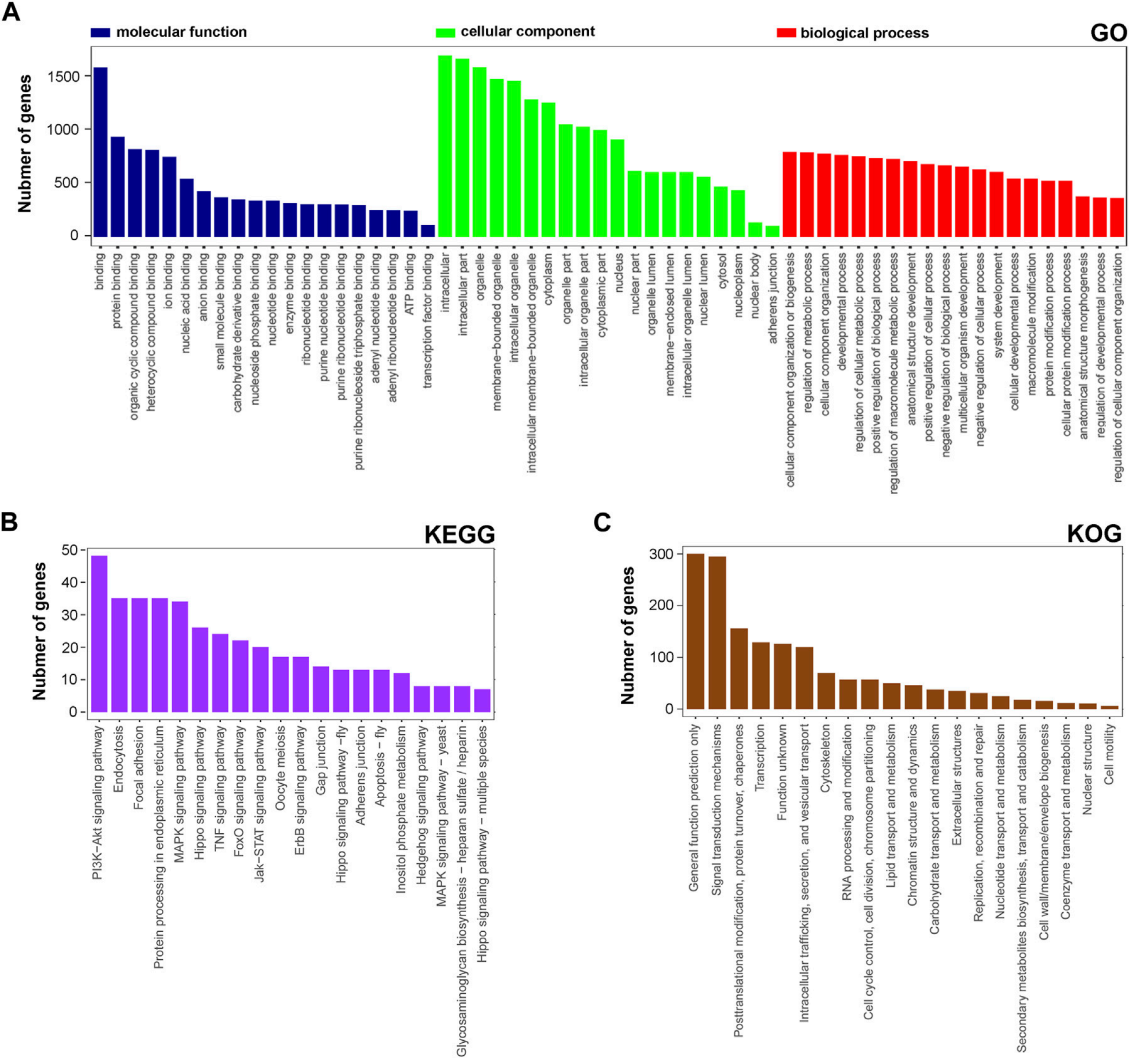
GO, KEGG and KOG analysis of differentially expressed genes DEGs based on transcriptomic data. GO **(A)**, KEGG **(B)**, and KOG **(C)** functional classification annotations. The *x*-axis represents the GO/KEGG/KOG category, and the *y*-axis represents the number of genes. Abbreviations: DEGs, differentially expressed genes; GO, Gene Ontology; KEGG, Kyoto Encyclopedia of Genes and Genomics; KOG, Nr eukaryotic orthologous groups.

Next, the functional distribution characteristics of the DEGs based on the GO, KEGG, and KOG annotations were obtained. In the GO annotation, each of the top 20 terms with significance from molecular function (MF), cellular component (CC), and biological process (BP) categories were presented using a histogram, and columns were sorted by the number of the enriched DEGs in that term (Figure 2A). The predominant two terms of each category were ‘binding’ and ‘protein binding’ in MF, ‘intracellular’ and ‘intracellular part’ in CC, and ‘cellular component organization of biogenesis’ and ‘regulation and metabolic process’ in BP. In the KEGG annotation, the predominant two terms were ‘PI3K-Akt signaling pathway’ and ‘endocytosis’ (Figure 2B). In the KOG annotation, the predominant two terms were ‘general function prediction only’ and ‘signal transduction mechanisms’ (Figure 2C). In addition, DEGs were subjected to KEGG and KOG network analyses. The main KEGG terms associated with the most dysregulated DEGs contained ‘focal adhesion’, ‘TNF signaling pathway’ and ‘PI3K-Akt signaling pathway’ (Supplementary Figure S1A). The KOG network contained main terms of ‘signal transduction mechanisms’, ‘transcription’ and ‘posttranslational modification, protein turnover, chaperones’ (Supplementary Figure S1B). According to the enrichment results, we found that lncRNA rPvt1 is associated with transcription, protein modification, signal transduction and other cellular behaviors, and links with PI3K/AKT pathway transduction.

### Quantitative proteomic analysis of lncRNA rPvt1 associations in LPS-treated H9C2 cardiomyocytes

**FIGURE 3:**
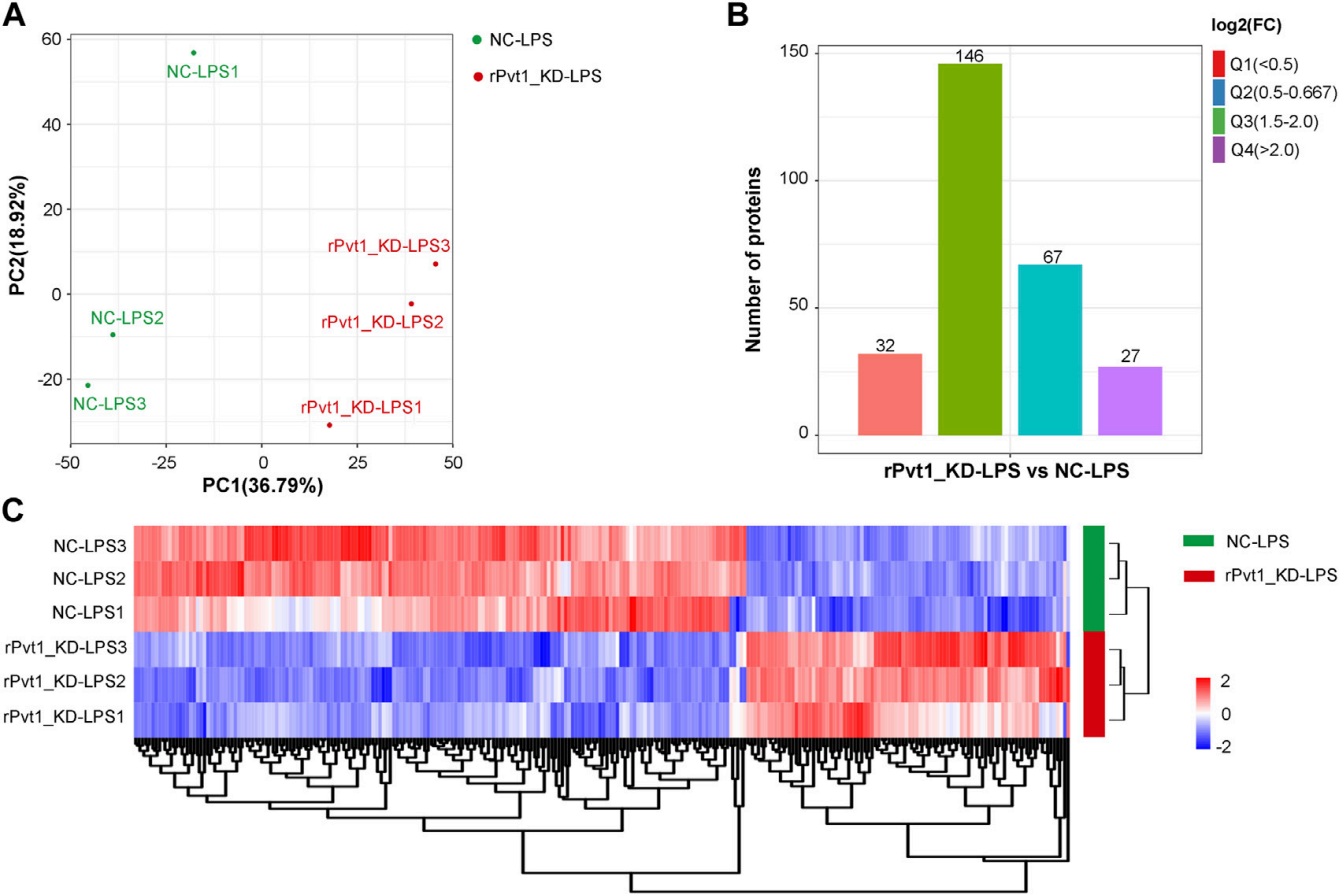
Quantitative proteomic analysis of lncRNA Pvt1 associations in lipopolysaccharide (LPS)-treated H9C2 cardiomyocytes. Rat H9C2 cardiomyocytes were infected with NC-shRNA harboring or rPvt1-shRNA harboring lentiviral particles for 96 h then treated with 10 μg/mL of LPS for 24 h. Quantitative proteomic analysis was established with three duplicated cell samples in each group. **(A)** PCA based on the protein abundance profile of each sample. **(B)** Distribution of the DAPs. Q1 (0<ratio<0.5), Q2 (0.5<ratio<0.667), Q3 (1.5<ratio<2), and Q4 (ratio>2); *p* < 0.05. **(C)** Heatmap of DAPs comparing NC-LPS and rPVT1_KD-LPS samples. Significantly upregulated and downregulated proteins are colored in red and blue, respectively. Abbreviations: DAPs, differentially abundant proteins; KD, knockdown; LPS, lipopolysaccharide; NC, negative control; PCA, principal component analysis; rPvt1, *Rattus norvegicus* lncRNA Pvt1; shRNA, short-harpin RNA.

As results of transcriptomic sequencing revealed lncRNA rPvt1 links with protein function, therefore, we investigated the proteome changes of rPvt1 silencing in LPS-treated H9C2 cardiomyocytes using a 4D label-free proteomic quantitative technique. PCA of all protein abundance revealed that the detected samples could be approximately divided into two categories (Figure 3A), suggesting the technical reproducibility of our data. In total, 5,281 proteins were identified, of which 4,305 were quantified. Among the DAPs, levels of 94 were 178 were upregulated and downregulated, respectively, in the rPvt1-silenced H9C2 cells after LPS treatment. The distribution of DAPs with different fold-changes is displayed as a column diagram (Figure 3B), and the overall abundance of DAPs is shown as a heatmap (Figure 3C). DAPs were determined to be mainly located in the nucleus, cytoplasm, mitochondria, plasma membrane, and extracellular space (Supplementary Figure S2A).

**FIGURE 4:**
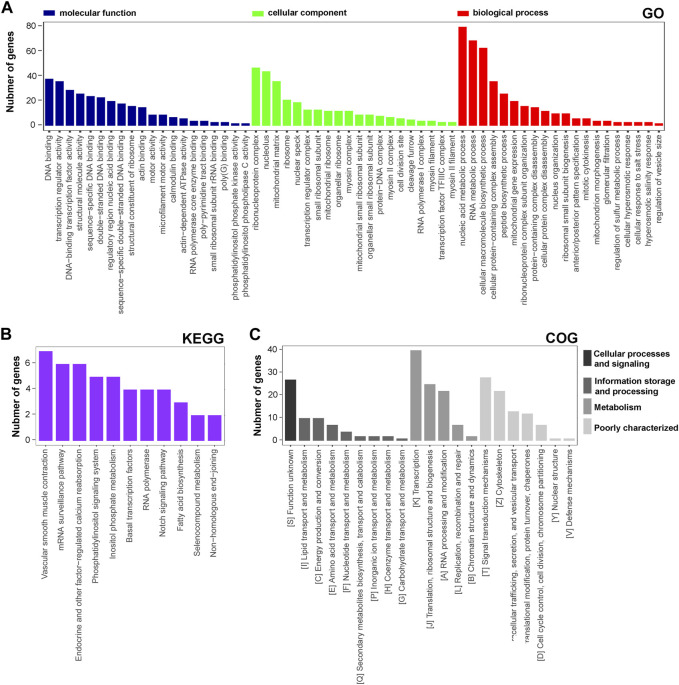
GO, KEGG and COG analysis of DAPs based on proteomic data. GO **(A)**, KEGG **(B)**, and COG **(C)** functional classification annotations. The *x*-axis represents the GO/KEGG/KOG category, and the *y*-axis represents the number of proteins. Abbreviations: DEGs, differentially expressed genes; GO, Gene Ontology; KEGG, Kyoto Encyclopedia of Genes and Genomics; COG, clusters of orthologous groups.

The functional distribution characteristics of the DAPs were allocated based on the GO, KEGG, and COG databases. Similar to transcriptomic analysis, each of the top 20 terms from MF, CC, and BP categories were selected and ranked by the enriched DAP number (Figure 4A). The main two terms in MF were ‘DNA binding’ and ‘transcription regulator activity’, in CC were ‘ribonucleoprotein complex’ and ‘nucleolus’, and in BP were ‘nucleic acid metabolic process’ and ‘RNA metabolic process’. Regarding the KEGG annotation, 12 significant terms were displayed, including the main terms ‘vascular smooth muscle contraction’ and ‘mRNA surveillane pathway’ (Figure 4B). In the COG annotation, the top 20 terms in categories of cellular processes and signaling, information storage and processing, metabolism, and poorly characterized were displayed, and the two predominant terms were ‘transcription’ and ‘signal transduction mechanisms’ (Figure 4C). In addition, a PPI network of the DAPs was generated based on a combined_score≥0.7 (Supplementary Figure S2B), which consisted of 126 nodes and 256 edges. *LOC679683* (degree = 20), *Mrpl16* (degree = 20), *Mrps11* (degree = 20), *Aatf* (degree = 18), and *Mrps17* (degree = 18) were the five hub central proteins identified in the PPI network. Notably, *Mrpl16*, *Mrps11* and *Mrps17* are genes that encode mitochondrial ribosomal proteins (Cheong et al., 2020). From our point of view, lncRNA rPvt1 may be associated with transcription, nucleic acid metabolism and mitochondrial functions.

### Widely-targeted metabolomic analysis and energy-targeted metabolomic analysis of lncRNA rPvt1 associations in LPS-treated H9C2 cardiomyocytes

**FIGURE 5:**
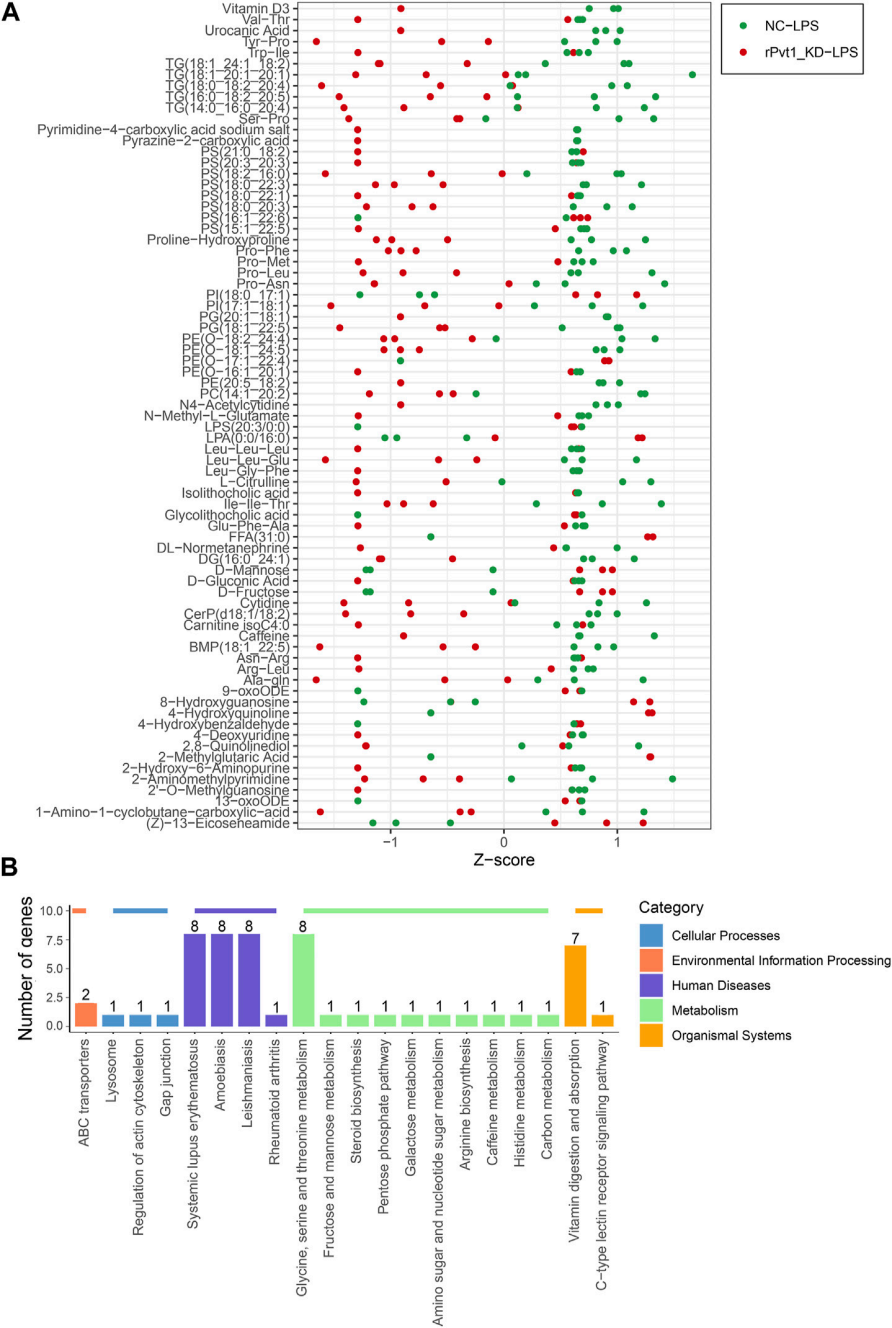
Widely targeted metabolomic analysis of lncRNA Pvt1 associations in LPS-treated H9C2 cardiomyocytes. Rat H9C2 cardiomyocytes were infected with NC-shRNA harboring or rPvt1-shRNA harboring lentiviral particles for 96 h then treated with 10 μg/mL of LPS for 24 h. Widely targeted metabolomic analysis was established with three duplicated cell samples in each group. **(A)** Z-score plot of the 75 DEMs. Each dot is a metabolite in each sample. Green dots represent metabolites in the NC-LPS group, and red dots represent metabolites in the rPVT1_KD-LPS group. **(B)** Enriched DEM-associated signaling pathways based on the KEGG database. Abbreviations: DEMs, differentially expressed metabolites; KD, knockdown; LPS, lipopolysaccharide; NC, negative control; rPvt1, *Rattus norvegicus* lncRNA Pvt1; shRNA, short-harpin RNA; KEGG, Kyoto Encyclopedia of Genes and Genomics.

Cell metabolism is an orderly series of chemical reactions that occur within cells. These reaction processes allow organisms to grow and reproduce, maintain their structure, and respond to the external environment. In this work, we conducted widely-targeted metabolomic analysis to investigate the cellular metabolites in LPS-treated H9C2 cardiomyocytes that regulated by lncRNA rPvt1. In total, 1,866 metabolites were analyzed, and 75 significant DEMs were identified after rPvt1 knockdown, of which levels of 59 were downregulated and 16 were upregulated. The Z-score plot showed that metabolism markedly differed between the two groups (Figure 5A). Hierarchical clustering of Pearson correlations showed correlations between DEMs. Most metabolites were positively correlated (Supplementary Figure S3). The altered metabolites were then subjected to KEGG database analyses. These DEMs were mainly enriched in ‘systemic lupus erythematosus’, ‘glycine, serine and threonine metabolism’, and ‘vitamin digestion and absorption’ (Figure 5B). Amino acids and vitamins are important energy sources and nutrients within cells. These results suggested that rPvt1 may be involved in energy metabolism and nutrition.

Furthermore, we conducted an energy-targeted metabolomic analysis. In total, 68 energy-related metabolites were analyzed, and 19 metabolites were found to be significantly changed, among which levels of all were downregulated. Similarly, a Z-score plot was generated to show the relative expression of DEMs (Supplementary Figure S4A), and a correlation heatmap suggested that all downregulated DEMs correlated with each other (Supplementary Figure S4B). KEGG enrichment analysis implied that the changes in these energy-related metabolites might be involved in the processes ‘aminoacyl-tRNA biosynthesis’, ‘alanine, aspartate and glutamate metabolism’, and ‘arginine biosynthesis’ (Supplementary Figure S4C). These amino acid metabolic pathways may be involved in the regulation of rPvt1 in cardiomyocyte function through participation in energy productivity.

### Integrated analysis of the transcriptomic and proteomic data

**FIGURE 6:**
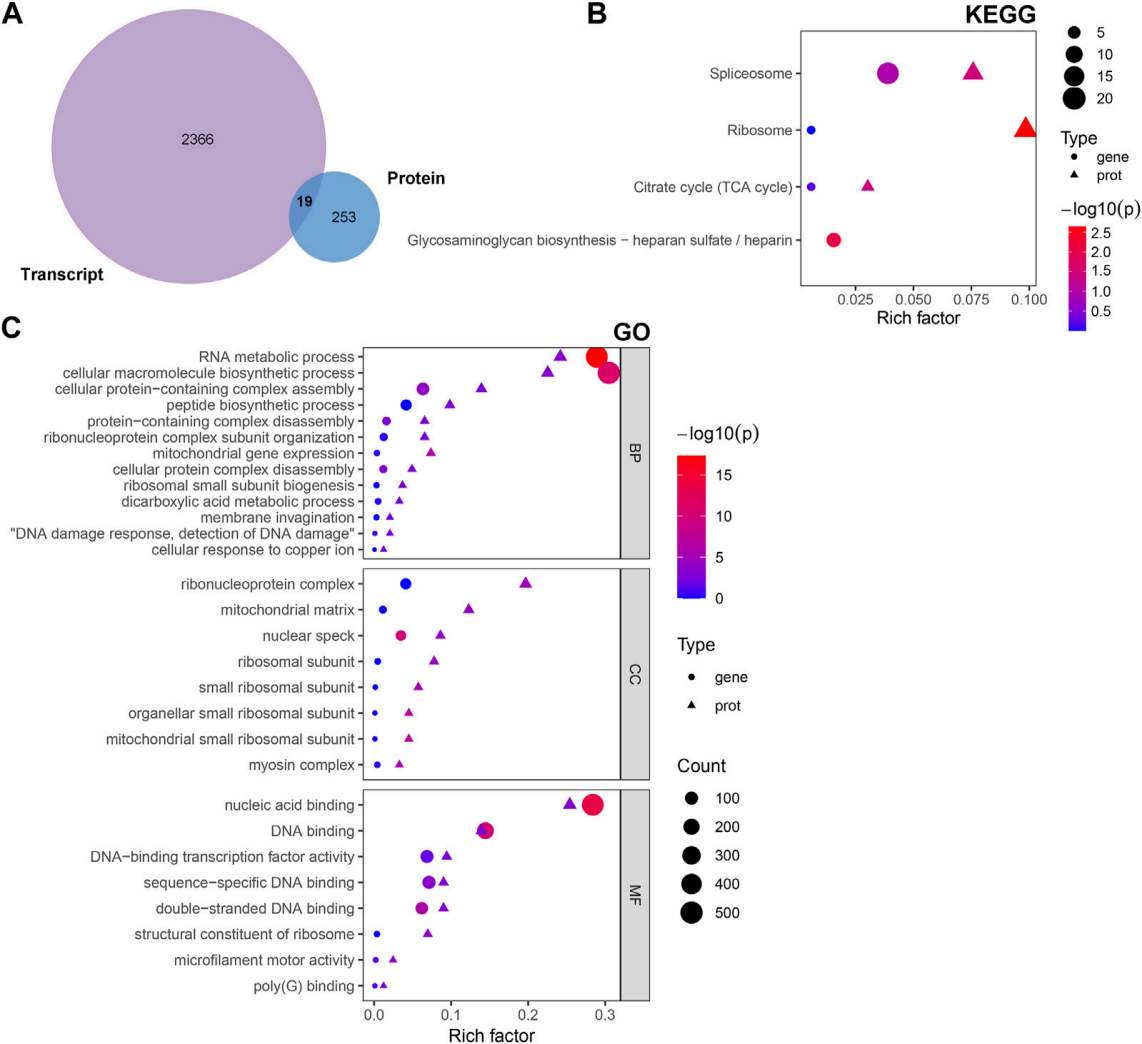
Integrated analysis of the transcriptomic and proteomic data. **(A)** Venn diagram representing the overlap between the DEGs and DAPs. Nineteen proteins/genes were commonly identified in both transcriptomic and proteomic profiling. **(B)** GO and **(C)** KEGG enrichment of DEGs/DAPs. Abbreviations: DAPs, differentially abundant proteins; DEGs, differentially expressed genes; GO, Gene Ontology; KEGG, Kyoto Encyclopedia of Genes and Genomics.

The multi-omics approach that integrates information of several omics, could provide more evidence for the biological mechanisms. Therefore, we conducted a joint analysis of every two omics in order to better understand the molecular mechanism of complex traits in biological and disease processes. First, we integrated transcriptomic and proteomic data. The DEGs and DAPs were next mapped based on their respective NCBI Gene Symbols, and overlapping genes/proteins between the transcriptomic and proteomic data were searched using a Venn diagram approach (Figure 6A). Nineteen common genes/proteins were identified, including *Cp, Tcerg1, Stam2, Cpsf6, Ddx42, Plcb3, Slit2, Hip1, Jak1, Pcyt1a, Aqp1, Ap2b1, Dars2, Cdc16, Atp2b4, Atp2a2, Hnrnpu, Tmlhe*, and *Extl3*. These genes were then subjected to GO and KEGG enrichment analyses. The KEGG analysis indicated that the common genes were enriched in the terms ‘ribosome’, ‘spliceosome’, ‘citrate cycle (TCA cycle)’, and ‘glycosaminoglycan biosynthesis - heparan sulfate/heparin’ (Figure 6B). Meanwhile, the GO analysis indicated that the common genes were enriched in ‘RNA metabolic process’, ‘ribonucleoprotein complex’ and ‘nucleic acid binding’ (Figure 6C). These pathways are mainly involved in the direction of energy productivity and metabolism.

### Integrated analysis of proteomic and metabolomic data

**FIGURE 7:**
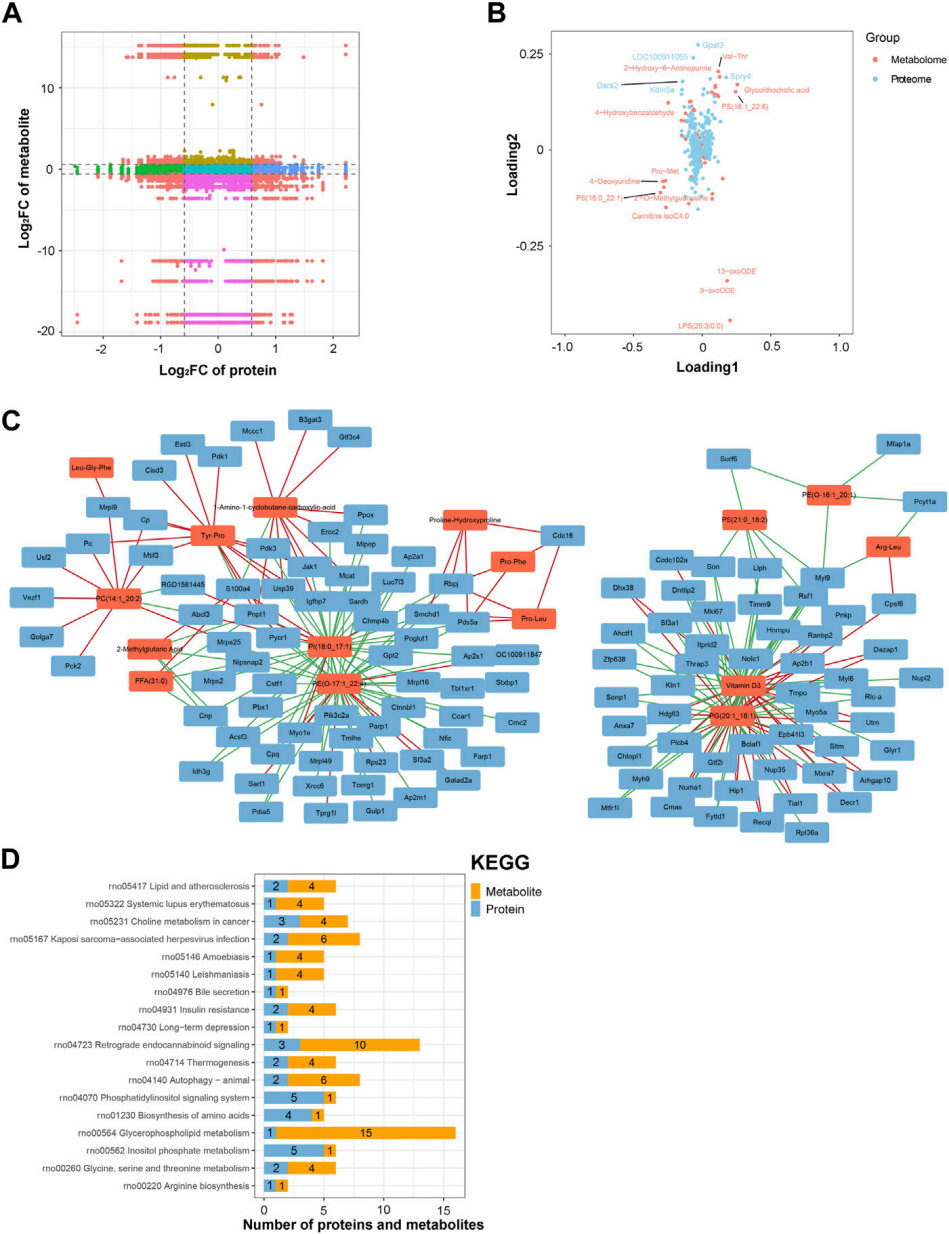
Integrated analysis of the proteomic and metabolomic data. **(A)** Nine-quadrant diagram representing the association between proteomic and metabolomic variations. **(B)** Top correlated DAPs and DEMs. Red dots represent metabolites, and blue dots represent proteins. **(C)** Network of the DAPs–DEMs. Red nodes represent metabolites, and blue nodes represent metabolites. **(D)** Correlated DAPs and DEMs involved in the KEGG pathway. Abbreviations: DAPs, differentially abundant proteins; DEMs, differentially expressed metabolites; KEGG, Kyoto Encyclopedia of Genes and Genomics.

An integrated analysis of the DAPs and DEMs was also conducted. The correlations between all DAPs and DEMs are presented using a clustering heatmap (Supplementary Figure S5). Then, the DAPs and DEMs with a correlation coefficient greater than 0.9 were selected and shown in a nine-quadrant diagram (Figure 7A). The third and seventh quadrants show DAPs and DEMs with consistent expression patterns, whereas the first and ninth quadrants show DAPs and DEMs with opposite expression patterns. The second, fourth, sixth, and eighth quadrants show instances of only one DAP or DEM exhibiting significant changes. Finally, the fifth quadrant shows DAPs and DEMs with no significant changes. Next, a loading diagram was plotted to show the correlated proteins and metabolites (Figure 7B). The top correlated proteins were *Gpat3, LOC100911055, Dars2, Spry4*, and *Kdm5a*, and the top correlated metabolites were Val-Thr, 2-hydroxy-6-aminopurine, PS (16:1_22:6), glycolithocholic acid, 4-hydroxybenzaldehyde, Pro-Met, 4-deoxyuridine, PS (18:0_22:1), 2-O-methylguanosine, carnitine isoC4:0, 13-oxoODE, 9-oxoODE, and LPS (20:3/0:0). A DAP–DEM network was further constructed to show their relationship (Figure 7C), and the correlated proteins and metabolic pathways were subjected to KEGG enrichment analysis. Twenty significant terms were identified, including the main term of ‘glycerophospholipid metabolism’ (Figure 7D). Glycerophospholipid metabolism is vital for biological membrane functions, fatty acids and carnitines, which are relevant to the energy production (Guo et al., 2022). Also, glycerophospholipid metabolism has been reported to be involved in the LPS-induced multiple organ injury (Wang et al., 2021). The results also suggested that energy metabolism may be involved in the regulation of lncRNA rPvt1 in LPS-treated cardiomyocytes.

### Integrated analysis of the transcriptomic and metabolomic data

**FIGURE 8:**
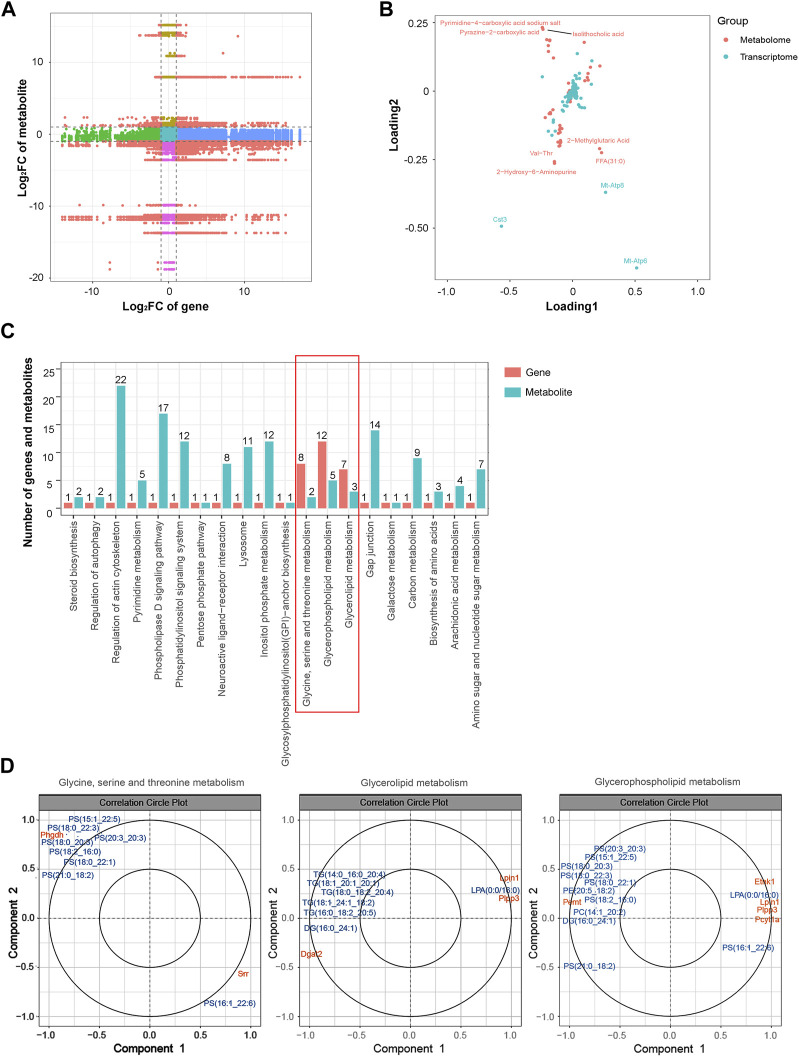
Integrated analysis of the transcriptomic and metabolomic data. **(A)** Nine-quadrant diagram representing the association between transcriptomic and metabolomic variations. **(B)** Top correlated DEGs and DEMs. Red dots represent metabolites, and blue dots represent genes. **(C)** Correlated DEGs and DEMs involved in the KEGG pathway. **(D)** CCA compares the DEGs and DEMs in the glycine, serine, and threonine metabolism; glycerolipid metabolism; and glycerophospholipid metabolism pathways. Gene IDs are in orange, and metabolite names are in blue. Abbreviations: CCA, canonical correlation analysis; DEGs, differentially expressed genes; DEMs, differentially expressed metabolites; KEGG, Kyoto Encyclopedia of Genes and Genomics.

Similarly, an integrated analysis of the DEGs and DEMs was conducted. The correlations between all DEGs and DEMs are presented using a clustering heatmap (Supplementary Figure S6). A nine-quadrant diagram was produced showing DEGs and DEMs with a correlation coefficient greater than 0.9 (Figure 8A). The loading diagram shows the correlated genes and metabolites (Figure 8B), including the genes *Cst3*, *Mt-Atp8*, and *Mt-Atp6* and the metabolites pyrimidine-4-carboxylic acid sodium salt, pyrazine-2-carboxylic acid, isolithocholic acid, Val-Thr, 2-methylglutaric acid, FFA (31:0), and 2-hydroxy-6-aminopurine. KEGG enrichment analysis was performed to show the correlated genes and metabolites, which were enriched in terms including ‘glycine, serine and threonine metabolism’, ‘glycerolipid metabolism’, and ‘glycerophospholipid metabolism’ pathways (Figure 8C). CCA diagrams further show the enriched genes and proteins in each pathway in detail (Figure 8D). Consistently, the enriched pathways were related to the mitochondrial energy metabolism.

## Discussion

Since their identification as epigenetic regulators, lncRNAs have been assumed to be associated with septic cardiac dysfunction (Li et al., 2021). Previous studies and those of our lab have demonstrated that the lncRNA Pvt1 is involved in cardiac injury after sepsis, but the associated mechanisms are largely unknown (Zhang et al., 2019; Luo et al., 2021). In the present study, we conducted transcriptomic, proteomic, and metabolomic assays to gain a better understanding of Pvt1 associations during LPS-induced injury using rat H9C2 cardiomyocytes.

Conventional transcriptome sequencing usually refers to RNA sequencing, which is an approach used to identify transcriptome profiles using deep-sequencing techniques (Lee et al., 2020). Compared with gene chip technology, RNA sequencing is not limited to complete genome data of the species. Moreover, it offers the advantages of high resolution, low background noise, high repeatability, and low cost (Head et al., 2014). Using RNA-sequencing techniques, we can not only rapidly obtain RNA expression levels and the functional annotation of DEGs but also discover new transcripts and structural variations (Zhao, 2019). To date, there have been hundreds of studies involving RNA-sequencing analysis that have been used to investigate the treatment and mechanism of septic myocardial injury (Xie et al., 2022; Yan et al., 2022). In this study, 2,385 DEGs were identified, of which levels of 2,094 were upregulated and those of 291 were downregulated. GO functional annotation revealed that the changes that occurred after rPvt1 silencing in LPS-treated H9C2 cells were associated with protein binding and cellular component organization. KEGG functional annotation further showed correlations with the regulation of the PI3K–Akt signaling and endocytosis pathways. The PI3K–Akt pathway is an important pathway that is closely associated with the pathological process of septic cardiomyopathy. The activation of this pathway delays LPS-induced inflammation, oxidative stress, and apoptosis induced by LPS (Li et al., 2019; Liu et al., 2021). Endocytosis is the process by which extracellular substances are transported into cells through deformable movement of the plasma membrane (Joshi et al., 2020). A previous study indicated that LPS must be internalized to promote endotoxin-dependent signaling in cardiomyocytes, and the internalization of LPS depends on endosomal transport. Accordingly, the inhibition of endocytosis specifically limits the early activation of extracellular signal-regulated kinase proteins and NF-κB, as well as the subsequent production of TNF-α and the expression of iNOS (Cowan et al., 2001). Other significantly enriched pathways, including focal adhesion (Davani et al., 2004), MAPK signaling (Frazier et al., 2012) and protein processing (Jiang et al., 2021) in the endoplasmic reticulum, are also related to septic myocardial dysfunction.

The emergence of MS-based high-throughput proteomic techniques, which increase the depth of protein coverage while reducing the sample analysis time, can quickly provide in-depth proteomic data and advance studies on septic myocardial dysfunction (Aslam et al., 2017). In this study, proteomic analysis revealed 272 significant DAPs in LPS-treated H9C2 cells following rPvt1 knockdown. Representative core proteins included three members of the mitochondrial ribosomal protein (MRP) family, specifically *Mrpl16, Mrps11*, and *Mrps17*. The PPI network further revealed a series of downregulated MRPs in the proteome. Mitochondria are the energy centers of cell (Andrieux et al., 2021). Because the heart requires large amounts of energy to sustain its continuous contractile activity, it is not surprising that mitochondria account for approximately 30% of the cardiomyocyte volume (Schaper et al., 1985). The metabolism of mitochondrial energy is essential for cardiomyocyte contraction and survival (Hasna et al., 2018). During septic cardiomyopathy, cardiomyocytes have been shown to exhibit ATP depletion and bioenergetic dysfunction (Curley et al., 2018). Also, the mitochondria are the main sites of ROS production and the main target of ROS attack and injury (Annesley and Fisher, 2019). In the LPS-injured heart, the excessive ROS easily injures mitochondria, leading to their dysfunction and energy metabolic shutdown (Stanzani et al., 2019). Intriguingly, the proteomic analysis related to these altered mitochondrial ribosomal proteins suggests that rPvt1 is associated with mitochondrial regulation in damaged cardiomyocytes. Functional enrichment analysis also demonstrated that mitochondria and ribosomes might be the main sites affected by rPvt1 modulation, suggesting the function of lncRNA rPvt1 in cardiomyocyte injury may be closely associated with the regulation of mitochondrial homeostasis.

Widely targeted metabolomics is a novel technology that integrates the advantages of the universality of non-target metabolomics and accuracy of targeted metabolomics technology. It has the characteristics of high throughput, ultrasensitivity, wide coverage, and qualitative and quantitative accuracy (Li et al., 2022). In this study, widely targeted metabolomics were used to detect 75 DEMs in LPS-treated H9C2 cells after rPvt1 knockdown, and levels of most of these DEMs were downregulated. Metabolomic pathway analysis revealed that energy- and catabolism–redox-related metabolic pathways were influenced by rPvt1 modulation. Furthermore, energy-targeted metabolomic analysis identified 19 energy-related DEMs of which levels were downregulated by rPvt1 knockdown, including amino acids, phospholipases, and nucleotides, indicating that rPvt1 might influence energy metabolism. Since mitochondria are the primary functional sites, these findings, interestingly, also suggest that the rPvt1 function may be involved in mitochondrial regulation.

Moreover, these integrated transcriptomic, proteomic, and metabolomic data provide in-depth insights into the complex mechanisms underlying rPvt1 associations. We conducted correlation analysis to determine the relationships among these three omics combinations. Integrative transcriptomic and proteomic analyses identified genes with significant changes at both the RNA and protein levels. These genes were determined to be mainly related to mitochondrial, ribosomal, and energy metabolic processes. Moreover, proteomic–metabolomic and transcriptomic–metabolomic analyses suggested that energy-related processes, such as TCA and glycerophospholipid metabolism, are involved in rPvt1 associations. The results of multi-omics integration further implicate that the function of rPvt1 in cardiomyocytes is related to energy regulation.

However, this study has some limitations. Firstly, a bigger cohort is required to validate and support our findings because our sample size was relatively small. Second, there were not enough *in vitro* or *in vivo* studies in our investigation to evaluate the expression levels of the identified genes, proteins or metabolites. This constraint might have diminished the precision and comprehensive understanding of our investigation. Further investigations utilizing a wider variety of experimental techniques and a larger and more varied sample size may shed further light on the function of lncRNA rPvt1 in septic myocardial injury.

## Conclusion

In summary, we used transcriptomic, proteomic, and metabolomic techniques to determine rPvt1 associations in LPS-treated cardiomyocytes. The results of the multi-omics analyses suggest that there are multiple regulatory mechanisms associated with rPvt1 in septic cardiomyopathy and that mitochondrial energy metabolism is one such possible mechanism. Exploring the effect of rPvt1 on mitochondrial energy metabolism might provide new insights into its use as a target to treat myocardial injury during sepsis.

## Data Availability

The datasets presented in this study can be found in online repositories. The names of the repository/repositories and accession number(s) can be found below: https://proteomecentral.proteomexchange.org/cgi/GetDataset?ID&equals;PXD043117.PXD043117.
