# Supplementary figures and images for: Integration of transcriptomic, proteomic, and metabolomic data to identify lncRNA rPvt1 associations in lipopolysaccharide-treated H9C2 cardiomyocytes
Source: Front Genet. 2023 Nov 29;14:1278830. doi: 10.3389/fgene.2023.1278830 (PMC10718647; doi:10.3389/fgene.2023.1278830)

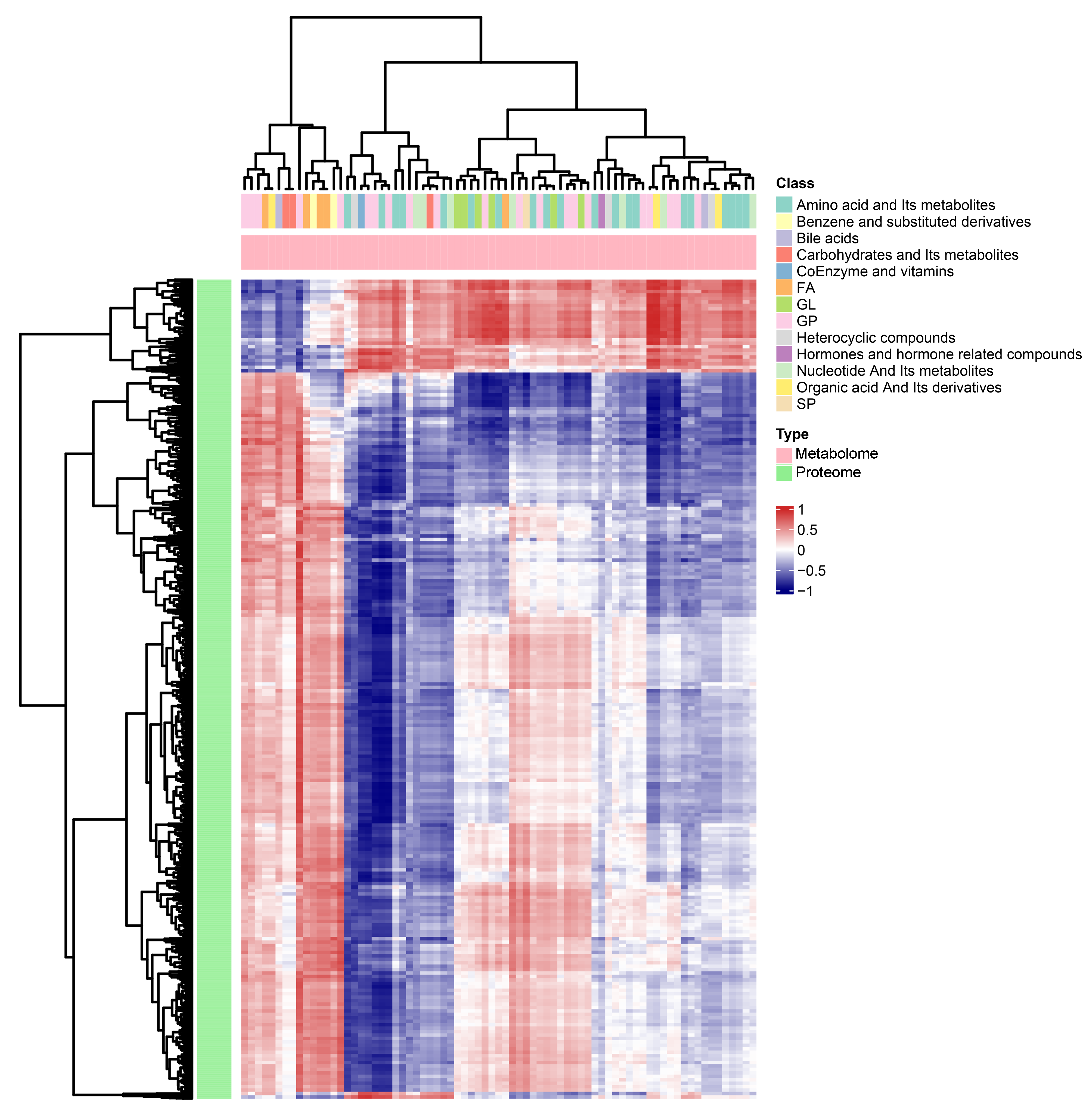

Supplement: Supplementary file 1 [file Image6.TIF]

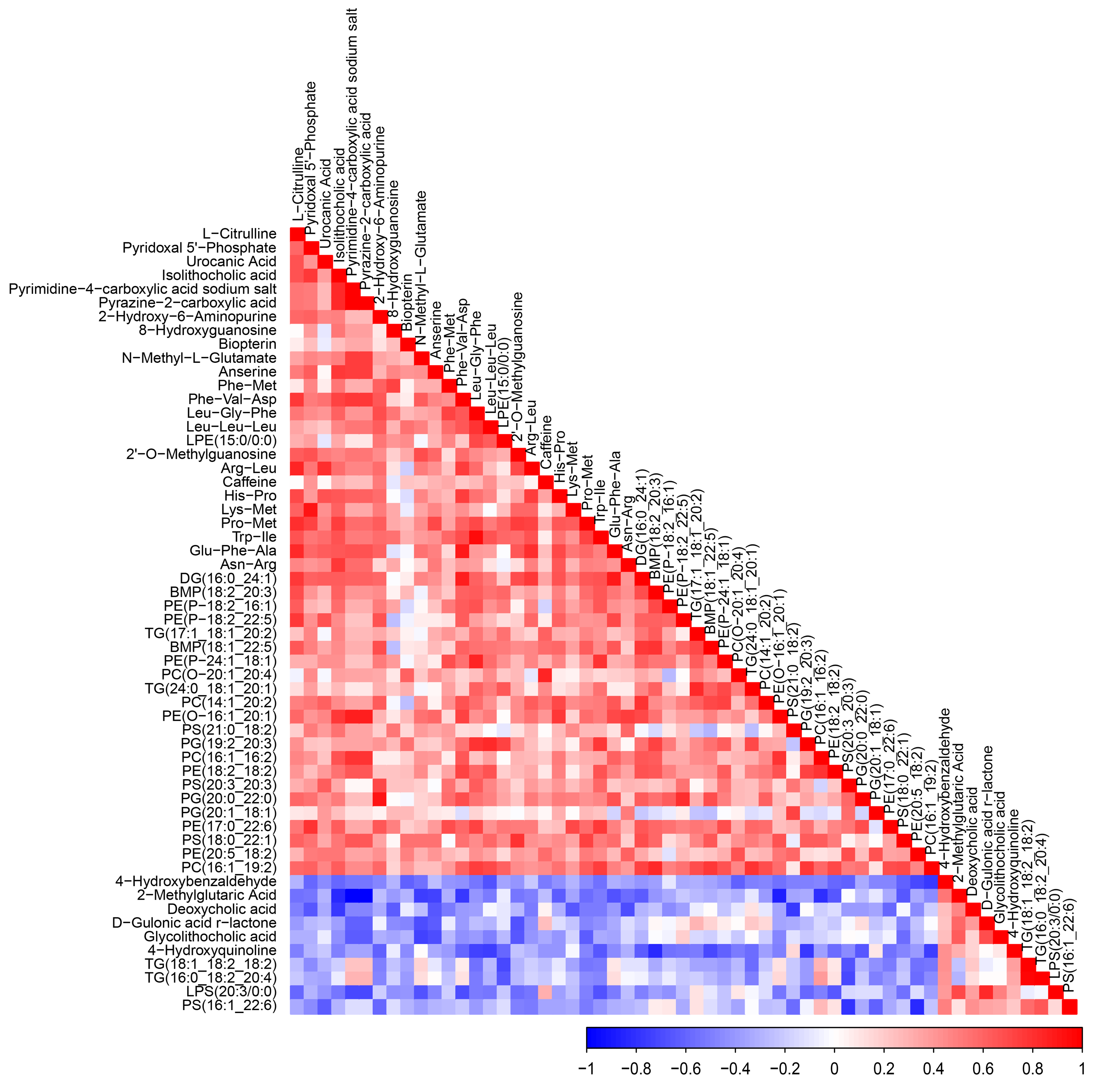

Supplement: Supplementary file 2 [file Image3.TIF]

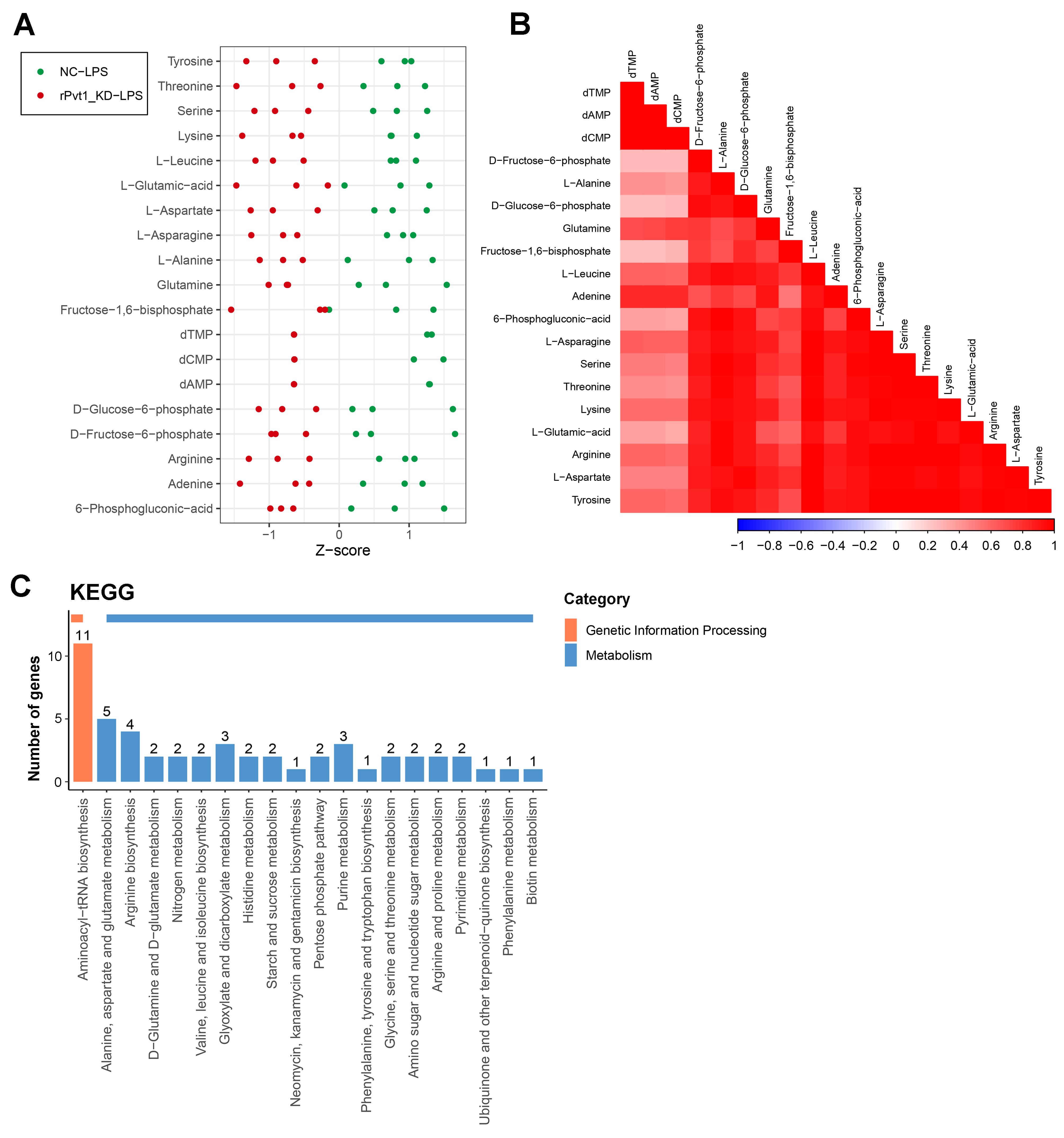

Supplement: Supplementary file 3 [file Image4.TIF]

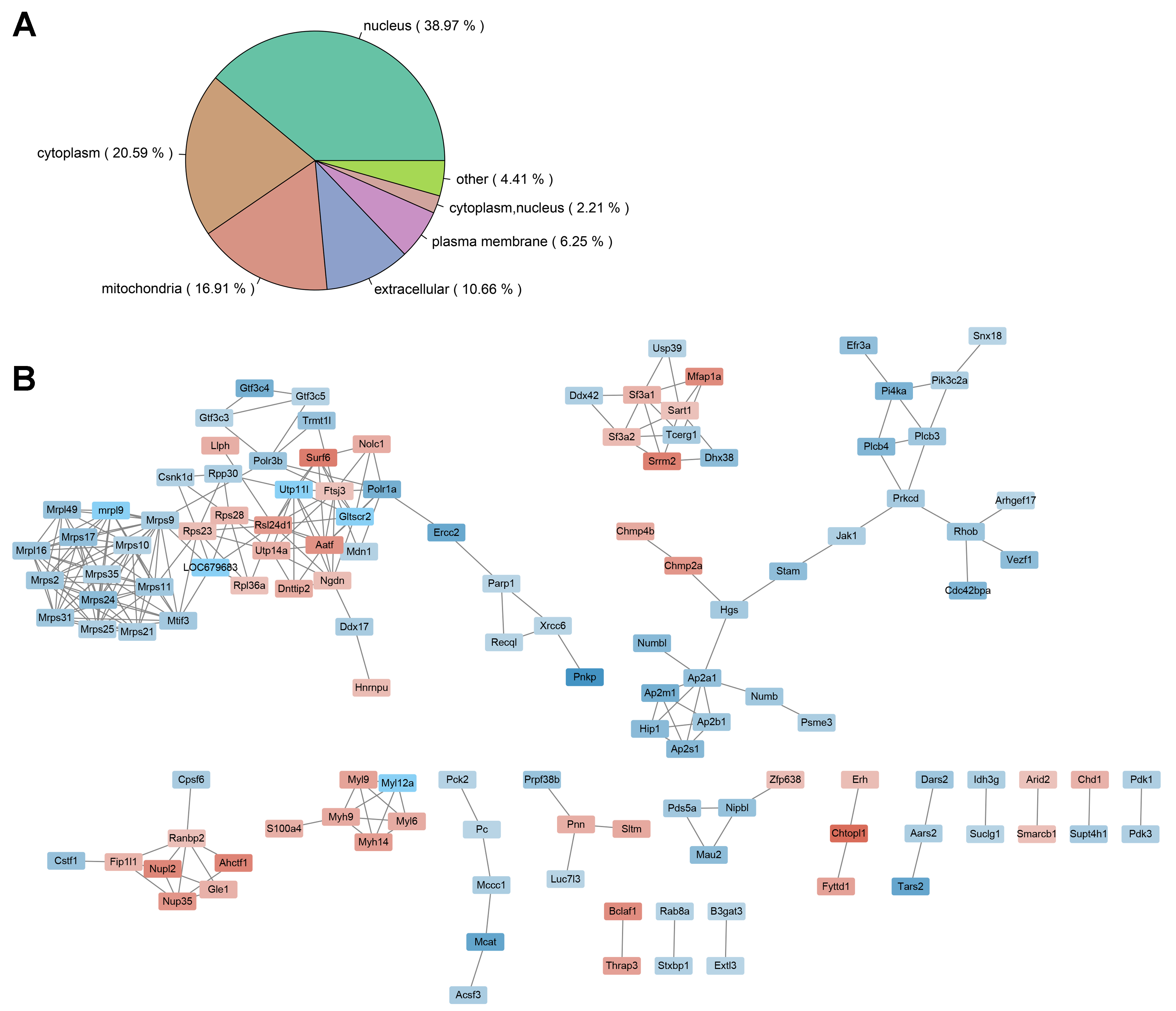

Supplement: Supplementary file 4 [file Image2.TIF]

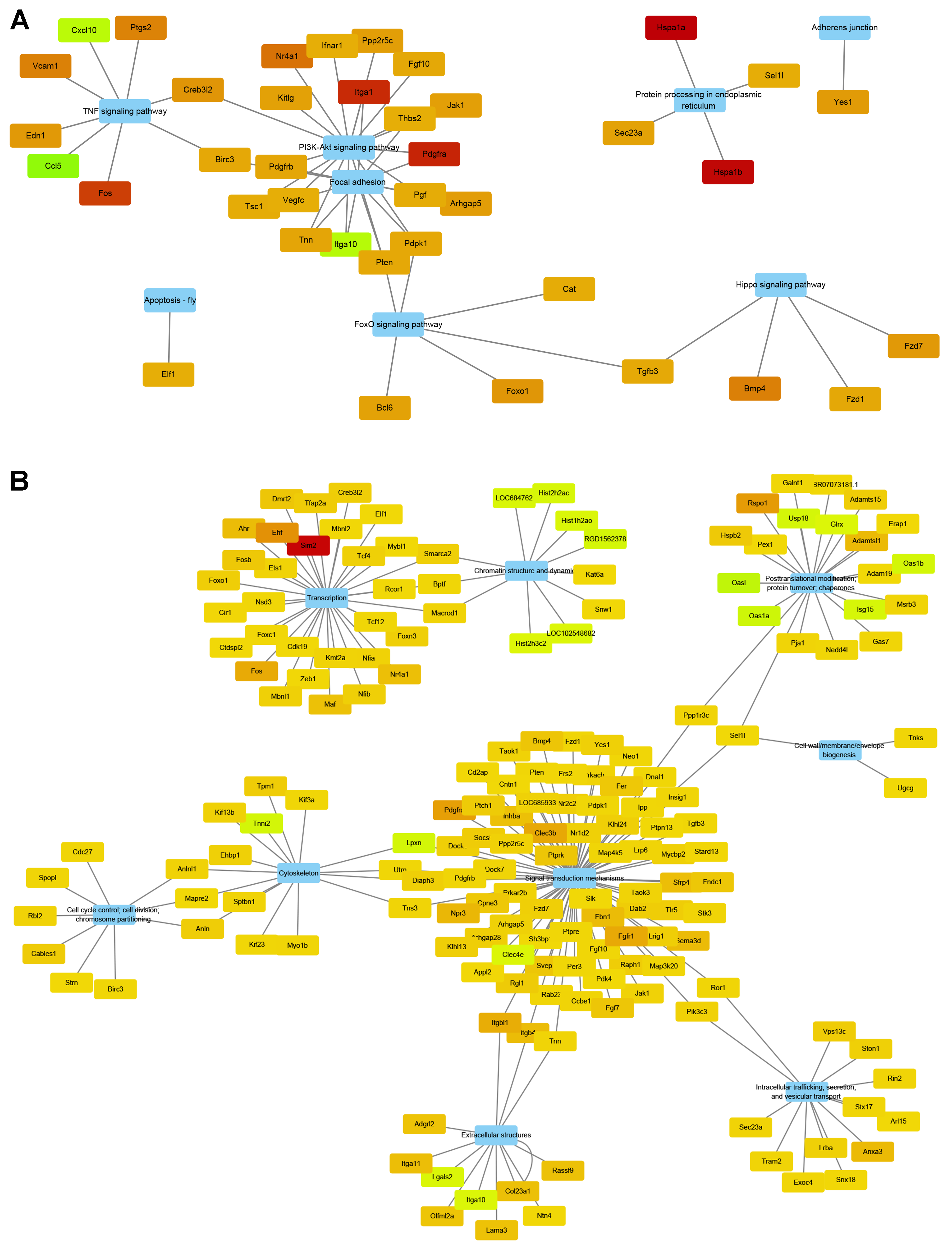

Supplement: Supplementary file 5 [file Image1.TIF]

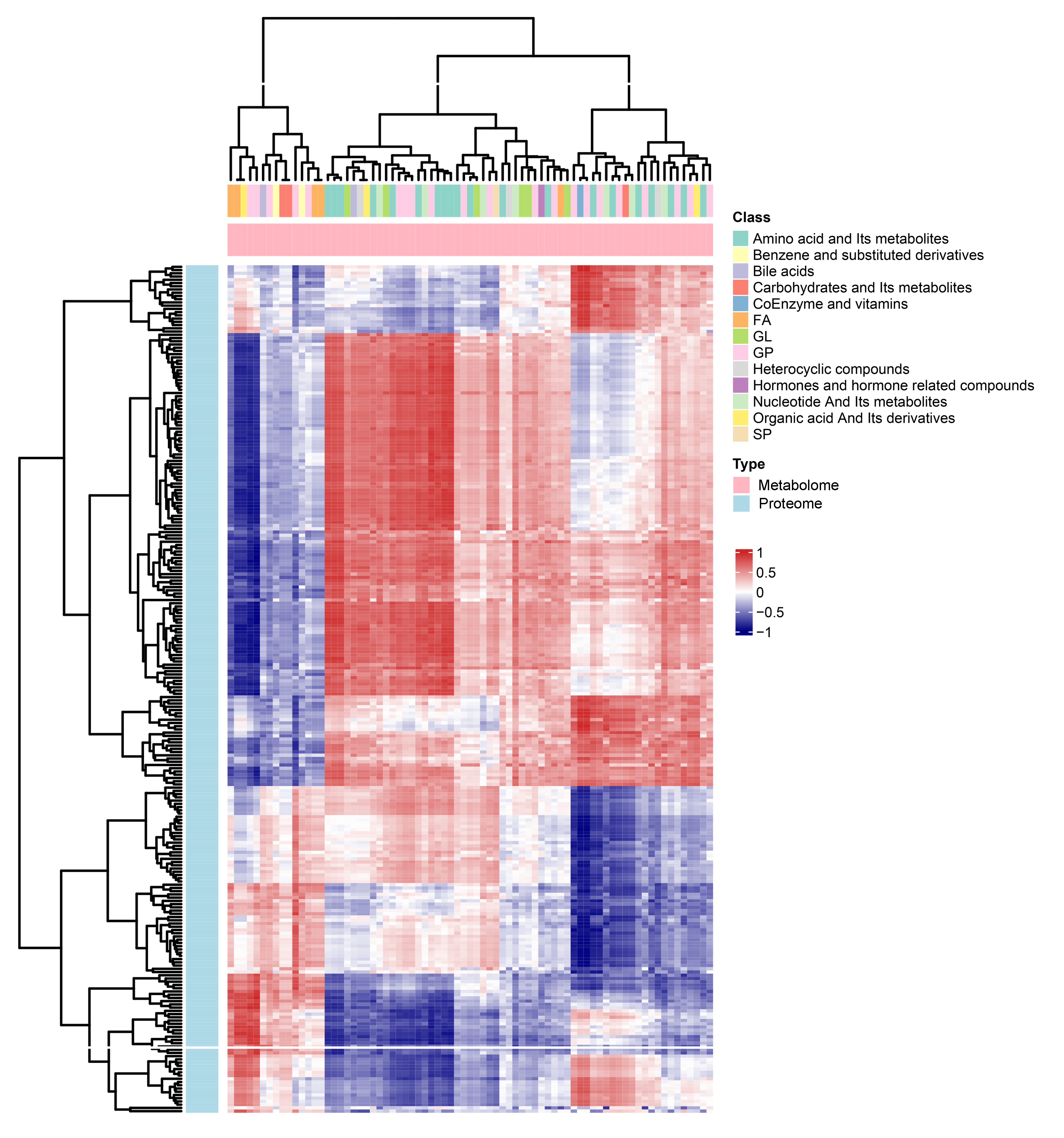

Supplement: Supplementary file 6 [file Image5.TIF]
